# List of reviewers in 2023

**DOI:** 10.1017/S0031182024000301

**Published:** 2024-03

## Abstract

**Figure:**
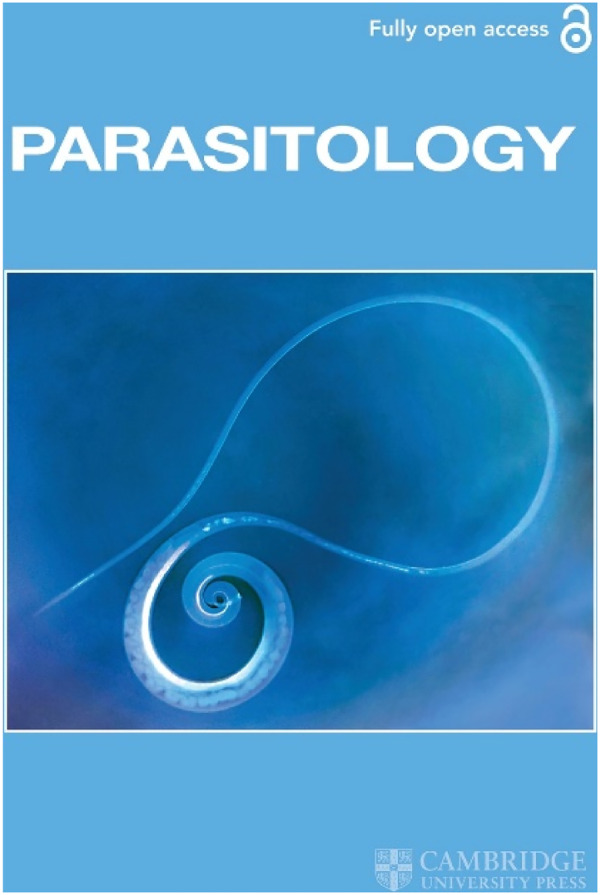

To publish an issue of *Parasitology* requires lots of help from “behind-the-scenes”. Over the last year, the journal successfully published 14 issues which collectively contain just over 150 articles in total. Two issues, numbers 12 and 14, were devoted to specialist topics originating from the Italian Society of Parasitology and Avian malaria theme, respectively.

In 2023, *Parasitology* successfully transitioned into full Open Access. This required several structural changes in the way manuscripts were handled through the publishing process yet one key activity, the peer-review process, remained unchanged. The editorial and publishing teams understand that reviewing a paper for *Parasitology* is no easy task.

Indeed, this year it remains a real privilege for our authors to have such expertise on call from just under 300 scientists from across the world. Thank you, once again, for making peer-review publication in *Parasitology* possible!

Here, we would like to take a moment to sincerely thank all our reviewers for their *pro bono* services to the journal in 2023, to our authors and to science in general.

Nidia Acosta, Instituto de Investigaciones en Ciencias de la Salud, Universidad Nacional de Asunción, Paraguay

Atheer H. Ali, University of Basrah, Iraq

Abdelghafar Alkishe, The University of Kansas, USA

Bassem Allam, Stony Brook University, USA

Paulina Alvarez Mendizabal, Universidad Nacional Autónoma de México Facultad de Medicina Veterinaria y Zootecnia, Mexico

Leucio Alves, Federal Rural University of Pernambuco, Brazil

Chrsitina Anaya, Florida Gulf Coast University, USA

Dmitry A. Apanaskevich, Georgia Southern University, USA

Rossana Arroyo, CINVESTAV-IPN, Mexico

Dmitry Atopkin, Federal Scientific Center of the East Asia Terrestrial Biodiversity, Russian Federation

Hamza Avcioglu, Ataturk University, Turkey

Carlos Azevedo, Institute of Biomedical Sciences Abel Salazar, University of Porto, Portugal

Francisco Javier Aznar, University of Valencia, Spain

Juan Antonio Balbuena, Universitat de València, Spain

Carlos Gustavo Baptista, University at Buffalo School of Medicine and Biomedical Sciences, USA

Iain Barber, Aberystwyth University, UK

Joel Barratt, Centers for Disease Control and Prevention, USA

Michael Begon, University of Liverpool, UK

Jeffrey Bell, University of North Dakota, USA

Michal Benovics, Masaryk University, Czech Republic

Ian Beveridge, University of melbourne, Australia

Natalia Biserova, University of Kansas, USA

David Blair, James Cook University, Australia

Tiffany Bouchery, Swiss Tropical and Public Health Institute, Switzerland

Geoffrey Boxshall, Natural History Museum, UK

Klaus Brehm, University of Würzburg, Germany

Laura Brettell, Liverpool School of Tropical Medicine, UK

Julia Buck, University of North Carolina at Wilmington Center for Marine Science, USA

Dovilė Bukauskaitė, Gamtos tyrimr centras, Lithuania

Richard Bungiro, Yale School of Public Health, USA

Francesco Buono, University of Naples Federico II, Italy

Alejandro Cabezas-Cruz, Ecole Nationale Veterinaire d'Alfort (ENVA), UK

Ivo Caldas, Universidade Federal de Alfenas, Brazil

Rafael Calero-Bernal, Complutense University of Madrid, Spain

Cyril Caminade, The Abdus Salam International Centre for Theoretical Physics (ICTP), Italy

Jianping Cao, Chinese Center for Disease Control and Prevention National Institute of Parasitic Diseases, China

Michael Cappello, Yale University, USA

David Carmena, Instituto de Salud Carlos III, Spain

Chris (Katharine C.) Carter, Strathclyde University, UK

Magali Casanova, Aix-Marseille Université, France

Rudi Cassini, Department of Animal Medicine, Production and Health, Italy

Serena Cavallero, Università degli Studi di Roma La Sapienza, Italy

Carolina Chagas, Nature Research Centre, Lithuania

Li Chao, Anhui Science and Technology University, China

Xuking Chen, Virginia Institute, USA

Arif Ciloglu, Erciyes Universitesi, Turkey

Graham Clark, London School of Hygiene and Tropical Medicine, UK

Joachim Clos, Bernhard Nocht Institut für Tropenmedizin, Germany

Eduardo Coelho, Universidade Federal de Minas Gerais, Brazil

Courtney Cook, North-West University, South Africa

Alba Cortes, Facultad de Farmacia-Universidad de Valencia, Spain

Sofia Cortes, Instituto de Higiene e Medicina Tropical, Portugal

Alice Costain, The University of Manchester, UK

Robert Cowie, University of Hawaii, USA

Thomas Cribb, University of Queensland, Australia

Charles Criscione, Texas A&M University, USA

Armando Cruz-Laufer, Hasselt University, Belgium

Scott Cutmore, University of Queensland, Australia

Krystyna Cwiklinski, University of Liverpool Institute of Infection Veterinary and Ecological Sciences, UK

Tad Dallas, Louisiana State University, USA

John Dalton, National University of Ireland Galway, Ireland

Paul Denny, Durham University, UK

Peter Deplazes, University of Zurich, Switzerland

Yves Desdevises, Universite Pierre et Marie Curie, Paris 6, France

Silvia Di Santi, Superintendencia de Controle de Endemias, Brazil

Stephen Doyle, Wellcome Sanger Institute, UK

Anna Dubiec, Polish Academy of Sciences, Poland

Meghan Duffy, University of Michigan, USA

Marcin Dziuba, University of Michigan, USA

Mostafa Elfawal, UMass Chan Medical School, USA

Claudia Esquivel, University of the Balearic Islands, Spain

Anna Faltynkova, Mendel University in Brno, Czech Republic

José Fernandez-Robledo, Bigelow Laboratory for Ocean Sciences, USA

Marcelo Ferreira, Institute of Biomedical Sciences, Brazil

Ivan Fiala, Biological Centre, AS CR, Czech Republic

Peter Fields, University of Basel, Switzerland

J Figuerola, EBD-CSIC, Spain

N Fraija, Universitat de Valencia, Spain

John Frean, National Institute for Communicable Diseases, South Africa

Brian Fredensborg, University of Copenhagen, Denmark

Caroline Frey, Canadian Food Inspection Agency, Canada

Bastian Fromm, SciLifeLab, Sweden

Holly Gaff, Old Dominion University, USA

Spencer Galen, University of Scranton, USA

Cristian Gallardo-Escárate, Universidad de Concepción, Chile

Terry Galloway, University of Manitoba, Canada

Rosana Gentile, Fundação Oswaldo Cruz, Brazil

Paul Giacomin, James Cook University Cairns Campus, Australia

Gary Gibson, Canadian National Collection of Insects Arachnids and Nematodes, Canada

Wendy Gibson, Bristol University, UK

John Gilleard, University of Calgary, Canada

Emma Gillingham, UK Health Security Agency, UK

Selma Giorgio, Biology Institute, University of Campinas UNICAMP, Brazil

Cameron Goater, University of Lethbridge, Canada

Geoffrey Gobert, Queen's University Belfast, UK

David Gonzalez, El Colegio De La Frontera Sur, Mexico

Angie González, Universidad Nacional de Colombia, Colombia

Simon Goodman, University of Leeds, UK

Catherine Gordon, QIMR Berghofer Medical Research Institute, Australia

Aditya Gupta, USDA-ARS Beltsville Agricultural Research Center, USA

Ankit Gupta, Central Drug Research Institute, India

Pooja Gupta, State of Utah, USA

Theresa Gyorkos, McGill University, Canada

Manuel Haimovici, Universidade Federal do Rio Grande, Brazil

Majid Fasihi Harandi, Kerman University of Medical Sciences, Iran (the Islamic Republic of)

Hanna Hartikainen, University of Nottingham Faculty of Medicine and Health Sciences, UK

Adam Hasik, Ben-Gurion University of the Negev, Israel

Hadas Hawlena, Ben Gurion University, Israel

Sara Healy, University of Surrey, UK

Hans Heesterbeek, Utrecht University Faculty of Veterinary Medicine, Netherlands

Andrew Hemphill, University of Bern, Switzerland

Michael Herman, University of Nebraska-Lincoln, USA

Carolina Hernández-Lara, Nature Research Centre, Lithuania

Robert Hirt, Newcastle University, UK

Jane Hodgkinson, University of Liverpool, UK

Amber Gigi Hoi, University of Ottawa, Canada

Celia Holland, University of Dublin, Trinity College, Ireland

Yoichiro Horii, University of Miyazaki, Japan

Laryssa Howe, Massey University, New Zealand

Yufei Huang, University of Dublin Trinity College, School of Business, Ireland

Abdul Jabbar, University of Melbourne, Australia

Fernando Jacinavicius, Instituto Butantan, Brazil

Joseph Jackson, University of Salford, UK

Susan Jarvi, University of Hawai'i at Hilo, USA

Gabriela Jeronimo, Universidade Federal do Amazonas, Brazil

Tiantian Jiang, University of California San Diego, USA

Francisco Jimenez-Ruiz, Southern Illinois University Carbondale, USA

Patricia Johnson, UCLA, USA

Pikka Jokelainen, Statens Serum Institut, Denmark

Alexandra Juhasz, Liverpool School of Tropical Medicine, UK

Patricia Kaishian, Bard College, USA

Ray Kaplan, University of Georgia, USA

Anssi Karvonen, University of Jyväskylä, Finland

Frank Katzer, Moredun Research Institute, UK

Jane Kelley, Australia Department of Primary Industries and Energy, Australia

Archie Khan, London School of Hygiene & Tropical Medicine, UK

Ruth Kirk, Kingston University, UK

Hans Klompen, Ohio State University, USA

Wayne Knee, Agriculture and Agri-Food Canada, Canada

Clemens Kocken, Biomedical Primate Research Centre, Netherlands

Heather Kopsco, University of Illinois at Urbana-Champaign, USA

Jules Kouadio, Centre Suisse de Recherches Scientifiques en Cote d'Ivoire, Côte d'Ivoire

Deborah Kristan, California State University San Marcos, USA

E. James La Course, Liverpool School of Tropical Medicine, UK

Kevin Lahmers, Virginia Polytechnic Institute, USA

Xavier Lambin, University of Aberdeen, UK

Erin Lashnits, University of Wisconsin-Madison, USA

Maria Latrofa, University of Bari, Italy

Matthieu Le Bailly, University of Bourgogne Franche-Comte, France

Jessica Light, Texas A&M University, USA

Marshall Lightowlers, The University of Melbourne, Australia

William Lin, Independent Scholar, USA

Tim Littlewood, Natural History Museum, UK

Guo-Hua Liu, Hunan Agricultural University, China

Claire Loiseau, Universidade do Porto Centro de Investigacao em Biodiversidade e Recursos Geneticos, Portugal

Ross Low, Earlham Institute, UK

Jasmina Ludoški, University of Novi Sad, Serbia

Luis Madeira de Carvalho, University of Lisbon, Portugal

Wanchai Maleewong, Faculty of Medicine, Khon Kaen University, Thailand

Carlos Martínez-Carrasco Pleite, Universidad de Murcia - Campus de Espinardo, Spain

Josué Martínez-de la Puente, University of Granada, Spain

Joaquina Martin-Sanchez, Granada University, Spain

Alfonso Marzal, University of Extremadura, Spain

Numair Masud, Cardiff University, UK

Alexander Mathis, University of Zurich, Switzerland

Sonja Matthee, Stellenbosch University, South Africa

Simonetta Mattiucci, Sapienza University of Rome, Italy

Maria Paola Maurelli, University of Naples Federico II, Italy

Milton McAllister, University of Adelaide, Australia

Rory McDonnell, Oregon State University, USA

Sergei Medvedev, Zoologiceskij institut RAN, Russian Federation

Naunain Mehmood, University of Sargodha, Pakistan

Jairo Alfonso Mendoza-Roldan, University of Bari, Valenzano, Italy, Italy

Andrei Mihalca, University of Agricultural Sciences and Veterinary Medicine Cluj-Napoca, Romania

Sergey Mironov, FSBIS Zoological Institute of the Russian Academy of Sciences, Russian Federation

David Modry, Czech University of Life Sciences Prague Faculty of Agrobiology Food and Natural Resources, Czech Republic

Sean Monaghan, University of Stirling, UK

Paul Monis, Australian Water Quality Centre, Australia

Scott Monks, Universidad Autonoma del Estado de Hidalgo Centro de Investigaciones Biologicas, Mexico

Antonio Montresor, WHO, Switzerland

Serge Morand, CNRS, France

Eric Morgan, Queen's University Belfast, UK

Russell Morphew, Aberystwyth University, UK

Grace Mulcahy, University College Dublin, Ireland

Sandrine Musa, University of Hohenheim, Germany

Lauren Nadler, University of Southampton, UK

Ryo Nakao, Hokkaido University, Japan

Abhijit Nandi, Guru Angad Dev Veterinary and Animal Sciences University, India

Rodrigo Narciso, São Paulo State University Institute of Biosciences, Brazil

Giuseppe Nascetti, Tuscia University, Italy

Elena Nassonova, Institute of Cytology RAS, Russian Federation

Gilmar Neves, Universidade Federal de São Carlos, Brazil

Chris Niebuhr, Landcare Research New Zealand, New Zealand

Marcelo Oliva, Universidad de Antofagasta, Chile

Francisco Olmo, Universidad de Granada, Spain

Peter Olson, The Natural History Museum, UK

Marketa Ondrackova, Institute of Vertebrate Biology, Czech Republic

Domenico Otranto, University of Bari, Italy

Hitoshi Otsuki, Tottori University Faculty of Medicine Graduate School of Medicine, Japan

Argun Akif Özak, Cukurova Universitesi, Turkey

Vaidas Palinauskas, Nature Research Centre, Lithuania

Camila Pantoja, Curso de Pós-Graduação em Ciências Veterinárias, Brazil

Barbara Paoletti, Faculty of Veterinary Medicine, University of Teramo, Italy

Gregorio Perez-Cordon, University of Granada Faculty of Pharmacy, Spain

Marie-Jeanne Perrot-Minnot, Université de Bourgogne, France

Giulio Petroni, University of Pisa, Italy

Mario Pinilla-Gallego, AGROSAVIA, Colombia

Hans Pohl, Friedrich Schiller University Jena, Germany

Petras Prakas, Nature Research Centre, Lithuania

Whitney Preisser, University of Washington, USA

Roger Prichard, McGill University, Canada

Iva Přikrylová, University of Limpopo, Turfloop Campus, South Africa

Meng Qi, Henan Agricultural University, China

R Rae, Liverpool John Moores University, UK

Amruta Rajarajan, Leibniz-Institute of Freshwater Ecology and Inland Fisheries in the Forschungsverbund Berlin eV, Germany

Haseeb Randhawa, University of Iceland, Iceland

Maria Teresa Reinoso-Perez, Cornell University, USA

Dania Richter, Technische Universität Braunschweig, Germany

Mark Robinson, Queen's University Belfast, UK

Michael Rogan, University of Salford, UK

Gemma Rojo, Universidad de O'Higgins, Chile

David Rollinson, The Natural History Museum, UK

Thomas Romig, University of Hohenheim, Germany

Bruce Rosa, Washington University School of Medicine in Saint Louis, USA

Andrew Rowley, Swansea University, UK

Sonja Rueckert, University of Duisburg-Essen, Germany

Urmas Saarma, University of Tartu, Institute of Ecology and Earth Sciences, Estonia

Harold Salant, Hebrew University of Jerusalem Robert H Smith Faculty of Agriculture Food and Environment, Israel

Maria Santos, Faculty of Sciences UP, Portugal

Davide Sassera, Università degli Studi di Pavia, Italy

Yukita Sato, Iwate University, Japan

Bahram Sayyaf Dezfuli, University of Ferrara, Italy

Andreas Schmidt-Rhaesa, Center of Natural History Leibniz Institute for the Analysis of Biodiversity Change, UK

Tomas Scholz, Institute of Parasitology, AS CR, Czech Republic

Marilyn Scott, McGill University, Canada

Otto Seppälä, University of Innsbruck, Austria

Jeffrey Shields, Virginia Institute of Marine, USA

Pavel Široký, University of Veterinary and Pharmaceutical Sciences, Faculty of Veterinary Hygiene and Ecology, Czech Republic

Jan Slapeta, University of Sydney, Australia

Brad E. E. Sleebs, University of Melbourne, Australia

John Smit, EIS Kenniscentrum Insecten, Netherlands

Nico Smit, North-West University, South Africa

Georges Snounou, UPMC/INSERM UMR S 945, France

Woon-Mok Sohn, Gyeongsang National Univresity College of Medicine, Korea (the Republic of)

Chinnaiyan Soundararajan, Tamil Nadu Veterinary and Animal Sciences University, UK

Jacek Sroka, National Veterinary Institute - National Research Institute, UK

Naima Starkloff, Emory University, USA

Peter Steinmann, Swiss Tropical and Public Health Institute, Switzerland

Russell Stothard, Liverpool School of Tropical Medicine, UK

Christina Strube, University of Veterinary Medicine Hannover, Germany

Xi Sun, Sun Yat-sen University, China

Ala Tabor, The University of Queensland, Australia

Kevin Tan, National University of Singapore, Singapore

Tiana Tasca, Universidade Federal do Rio Grande do Sul, Brazil

Samuel Teixeira, Universidade Federal de Uberlândia, Brazil

Richard Thompson, Murdoch University, Australia

Juan Timi, Universidad Nacional de Mar del Plata Facultad de Ciencias Exactas y Naturales, Argentina

Vasyl Tkach, University of North Dakota, USA

Rafael Toledo, Facultad de Farmacia-Universidad de Valencia, Spain

Paul Torgerson, Vetsuisse Faculty at the University of Zurich, Switzerland

Joseph Turner, Liverpool School of Tropical Medicine, UK

Kevin Tyler, University of East Anglia, UK

Geral Umhang, Anses Rabies and Wildlife Laboratory, France

Julio Urbina, Instituto Venezolano de Investigaciones Cientificas, Venezuela (Bolivarian Republic of)

Andrea Valigurová, Masaryk University, Faculty of Science, Czech Republic

Gediminas Valkiūnas, Nature Research Centre, Lithuania

Lisette van Lieshout, Leiden University Medical Center, Netherlands

Antonio Varcasia, Università degli Studi di Sassari, Italy

Jefferson Vaughan, University of North Dakota, USA

Vincenzo Veneziano, University of Naples Federico II, Italy

Fabrizia Veronesi, University of Perugia, Italy

Jaco Verweij, Elisabeth-TweeSteden Ziekenhuis, Netherlands

Petr Volf, Charles University, Czech Republic

Georg von Samson-Himmelstjerna, Freie Universität Berlin, Germany

Chun-Ren Wang, Heilongjiang Bayi Agricultural University, China

Elizabeth Warburton, University of Georgia, USA

Marion Wassermann, University of Hohenheim, Germany

Matthew Wayland, University of Cambridge, UK

Andreia Wendt, University of Bern, Switzerland

Marius Wenzel, University of Aberdeen, UK

Alan Wilson, University of York Department of Biology, UK

Radosław Włodarczyk, University of Lodz Faculty of Biology and Earth Sciences, Poland

Chengzhong Yang, Chongqing Normal University, China

Ana Yatsuda, Universidade de Sao Paulo, Brazil

Vyacheslav Yurchenko, University of Ostrava, Czech Republic

Patricia Zajaczkowski, University of Technology Sydney, Australia

Longxian Zhang, Henan Agricultural University, China

Annetta Zintl, University College Dublin, Ireland

